# Changes in Antioxidants and Sensory Properties of Italian Chocolates and Related Ingredients Under Controlled Conditions During an Eighteen-Month Storage Period

**DOI:** 10.3390/nu11112719

**Published:** 2019-11-09

**Authors:** Arianna Roda, Milena Lambri

**Affiliations:** DiSTAS—Department for Sustainable Food Process, Università Cattolica del Sacro Cuore, 29122 Piacenza, Italy; arianna.roda@unicatt.it

**Keywords:** Italian chocolate, quality, cocoa-based ingredients, monitoring, nutrition

## Abstract

Background: While there has been an increasing interest in the health properties of chocolate, limited research has looked into the changes of antioxidants occurring in the time span from production to the best before date, which was a period of 18 months in this study. Methods: Humidity, ash, pH, acidity, fiber, carotenoids, retinols, tocopherols, sugars, proteins, theobromine, caffeine, polyphenols, fats, the peroxide value, organic acids, and volatile compounds, along with the sensory profile, were monitored at 18-week intervals for 18 months under conditions simulating a factory warehouse or a point of sale. Results: At the end of the storage period, more polyphenols were lost (64% and 87%) than vitamin E (5% and 14%) in cocoa mass and cocoa powder, respectively. Conversely, a greater loss in vitamin E (34% and 86%) than in polyphenols (19% and 47%) was shown in the hazelnut paste and gianduja chocolate, respectively. The sensory profiling of cocoa mass, cocoa powder, and hazelnut paste revealed increases in grittiness and astringency, as well as decreases in melting, bitterness, and toasted aroma. Moreover, in the hazelnut paste and gianduja chocolate, oiliness increased with a toasted and caramel aroma. Furthermore, dark chocolate was more gritty, acidic, and bitter. Milk chocolate lost its nutty aroma but maintained its sweetness and creaminess. Conclusions: These results should contribute an important reference for companies and consumers, in order to preserve the antioxidants and understand how antioxidants and sensory properties change from the date of production until the best before date.

## 1. Introduction

Many daily consumed foods, such as fruits, vegetables, wine, coffee, and chocolate, are attracting increasing attention due to their potential health effects thanks to their richness in polyphenols [1,2]. Recent studies have focused on cocoa products commonly consumed for pure pleasure, since they also generate tangible benefits for human health [3]. Indeed, cocoa is a complex product with over 300 constituents [4] and one of the richest sources of flavanols [5]. Cocoa belongs to the “nervine food group” because it contains xanthine alkaloids, with the most important being theobromine (2%) and caffeine [6]. When eaten in moderation as part of a balanced diet, it has been suggested that flavanols from cocoa products may exert beneficial effects on the cardiovascular risk via effects of lowering the blood pressure, anti-inflammation, antiplatelet function, higher HDL, and decreased LDL oxidation. This evidence has been supported by a systematic review of 136 publications [7] and by the European Food Safety Agency [8], which stated a health claim for dark chocolate with a high flavanol content due to its impact on “maintenance normal endothelium-dependent vasodilation which contributes to normal blood flow”. In order to obtain the claimed effect, 200 mg of cocoa flavanols should be consumed daily. This amount could be provided by 2.5 g of high-flavanol cocoa powder or 10 g of high-flavanol dark chocolate.

On the other hand, the popularity of hedonic foods, like chocolate, mostly depends on its sensory properties, which in turn constitute the key for the acceptance of food products on the market. Consumers measure the quality and take raw information on the composition while evaluating the appearance, aroma, texture, taste, and flavor [9,10]. Additionally, experienced consumers have their own capability to search for defects leading to the rejection of chocolate and often caused by factors such as the temperature, relative humidity, and light [11], which in turn affect the stability of cocoa butter [9,12,13]. Indeed, the fat components are the main components responsible for oxidative deterioration which leads to the formation of off-flavors in chocolates [9,14].

Although there has been an increasing interest in the health properties of chocolate, mainly due to its polyphenol content, limited research has looked into the chocolate matrix to investigate the changes in polyphenols, vitamins, fats, and volatile compounds which also determine the quality [15,16,17]. While consumers are increasingly demanding high-quality food, they expect this quality to be maintained until the consumption time, but little is known about the evolution of chocolates and related raw materials in response to the storage conditions in a factory warehouse, as well as at a point of sale. The present study aims to monitor Italian chocolates and related ingredients for their polyphenol concentration, vitamin E, peroxide, and acidity values during storage for eighteen months under controlled conditions simulating a factory warehouse or a point of sale. In this study, sensory analysis was considered to outline the chemical changes which produce wider variations, so is more interesting from a commercial point of view.

## 2. Materials and Methods

### 2.1. Samples

Three types of chocolate (dark (D), milk (M), and gianduja (G)) and three ingredients (cocoa mass in solid form (CM), cocoa powder 22–24 (C), and hazelnut paste (HP)) were supplied by Venchi S.p.A. (Castelletto Stura, Cuneo, Italy). The recipes of the chocolates were as follows:

- D: cocoa 56% min., cocoa mass, sugar, cocoa butter, emulsifier: soy lecithin, and natural vanilla flavor;
- M: cocoa 31.8% min, milk: 23.5% min., sugar, whole milk powder, cocoa butter, cocoa mass, anhydrous milk fat, emulsifier: soy lecithin, and natural vanilla flavor;
- G: cocoa 21.2% min., Piedmont hazelnut pasta I.G.P. (33%), sugar, whole milk powder, cocoa butter, cocoa mass, anhydrous milk fat, emulsifier: soy lecithin, and natural vanilla flavor.

All the samples were collected immediately after production (t0) and stored in their own packages in an air-conditioned room at 21 ± 2 °C and 65% Relative Humidity (RH) for eighteen months, i.e., the best before date established by the company within the EU Regulation 2073:2005 [18]. An eighteen-week interval was decided for sampling considering both the number of samples made available by Venchi S.p.A and the storage time (t1, t2, t3, and t4).

### 2.2. Microbiological Analysis

The total microbial count (bacteria, fungi/molds, and yeasts) according to International Standard Organization (ISO) 4833-1:2013 [19], along with *Enterobacteriaceae* and coliforms as respectively described in ISO 21528-1:2017 [20] and ISO 4832:2006 [21], were detected at the production time (t0) and at the end of storage (t4). The number of colony-forming units (CFU) per gram of dry mass of the sample was provided.

### 2.3. Chemical Analysis

#### 2.3.1. Humidity, Ash, pH, Acidity, and Fiber

Humidity and ash were determined according to Association of Official Analytical Chemists (AOAC) 931.04 [22], while food and raw fiber were analyzed using the AOAC 985.29 [23] methodology. Total acidity and pH were analyzed as reported in the official methods of the Office International du Cacao et du Chocolat et de la Confiserie [24]. For both analyses, a pH meter (CRISON MICRO TT 2050, Carpi, Modena, Italy) was used. The pH of a 10 g sample dissolved in 90 mL of boiling distilled water was measured. The acidity was dosed in the same solution with 20 mL of sodium hydroxide 0.1 N and then potentiometrically titrated with hydrochloric acid 0.1 N until pH 7.0. The result was finally expressed in mg equivalents of stearic acid in a 100 g sample.

#### 2.3.2. Carotenoids, Retinol, Tocopherols, and Sugars

Carotenoids and retinol were determined by applying the AOAC 941.15 method [25], while tocopherols were dosed in compliance with Calvo et al. [26] and Belšcak et al. [27]. The chromatographic determination of tocopherols was performed on an HPLC system, including a Perkin Elmer (Norwalk, CT, USA) 200 Series pump equipped with a Perkin-Elmer 650-10S fluorescence detector, Jasco LC-Net II/ADC (Oklahoma City, OK, USA) communication module, and ChromNAV Control Center software. A LiChrosorb Si60-5 C18 column 250 mm × 4.6 mm, 5 µm (Supelco, Bellefonte, PA, USA) was used, the mobile phase was hexane:isopropanol:ethanol (98.5:1:0.5) at a flow rate of 1.0 mL/min and the injection volume was 20 µL. The fluorescence detector was set at 290 nm excitation and 330 nm emission wavelengths. α-, γ-, and δ- tocopherols were identified by comparing the retention times with those of commercial standards. The results were expressed as mg of Vitamin E including α-, γ-, and δ- tocopherols contained in 100 g of dry sample. To analyze reducing and non-reducing sugars in cocoa liquor and hazelnut paste, the Luff-Schoorl volumetric analysis suggested by Balestrieri and Marini [6] was adopted.

#### 2.3.3. Proteins

Protein determination was carried out following AOAC 939.02-1939 [28], as follows. Two grams of sample were dried in a stove for 24 h. Thirty milliliters of concentrated sulfuric acid (96%) were added to the sample and homogeneously dispersed, and the mixture was subsequently put into the digester (K-424 BUCHI) until complete digestion. Then, distillation was performed using a semi-automatic system (VELP SCIENTIFICA UDK R7), which adds 90 mL of sodium hydroxide (32%) and collects the distilled ammonia in 50 mL of boric acid (40 g/L), to which 0.5 mL of mixed indicator methyl red/bromocresol green was added. Finally, titration with 0.1 N sulfuric acid was carried out.

#### 2.3.4. Theobromine, Caffeine, and Polyphenols

For determining theobromine, caffeine, and polyphenols, samples were defatted by extracting 50 g of each sample three times with 250 mL of *n*-hexane and drying the resulting powder under a nitrogen stream to remove the residual organic solvent [29]. For determinations of caffeine and theobromine, the powder was dispersed in methanol before spectrophotometric analysis, which was carried out by means of a Lambda Bio 40 UV-VIS spectrometer (Perkin Helmer).

The theobromine was determined following López-Martìnez et al.’s method [30]. Since the maximum theobromine absorption in our samples was observed at 240 nm, a calibration curve with methanolic solutions containing 5, 10, 15, 20, 25, 30, and 35 mg/L of theobromine was produced at 240 nm.

Caffeine was determined according to the procedure described by Hečimović et al. [31]. The absorbance was read at 274 nm and a calibration curve was built using solutions at 20, 40, 60, 80, and 100 mg/L of caffeine dissolved in methanol.

The total polyphenol content was determined for the defatted samples dispersed in distilled water according to Belšcak et al. [27], and the absorbance was recorded at 765 nm using a Shimadzu UV-1601 spectrophotometer (Shimadzu Europe, Duisburg, Germany). Gallic acid was used as a standard in a calibration curve with solutions of 20, 40, 80, 100, and 120 mg/L of gallic acid. The results were expressed as mg gallic acid equivalents (GAE) per 100 g of defatted sample.

#### 2.3.5. Fat Content

To determine the fat content, the AOAC Official Method [25], modified as follows, was used. The samples initially underwent acid hydrolysis and extraction by means of a Soxhlet device. Different quantities of sample were weighed, including 3–4 g of cocoa liquor and hazelnut paste, and 4–5 g of cocoa powder. These quantities were mixed with 45 mL of distilled water at boiling point, after which 55 mL of hydrochloric acid (25% *w*/*v*) was added. The solutions boiled for about 30 min with a reflux condenser and were then filtered with a Whatman *n*° 595 ½ filter. The filter containing the hydrolyzed sample was thoroughly washed with distilled water until the chloride vanished, and it was then dried at 100 °C for 6 h. Finally, the fat matter was extracted by means of a Soxhlet device, where the dry filter underwent extraction with 50 mL of *n*-hexane for 4 h. The fatty acid and sterol profile were determined according to EC Regulation 2568/91 [32].

#### 2.3.6. Peroxide Value

To separate the lipid fraction, 50 g of each sample was extracted three times with 250 mL of *n*-hexane [29]. The resulting mixture was centrifuged at 3000 rpm for 15 min (Varifuge 20 RS Hereaus Sepatech, Hanau, Germany), and the hexane was removed using Rotavapor (Büchi Rotavapor R-114, Flawil, Switzerland). The oil recovered was analyzed to determine the peroxide value using the method reported in the EC Regulation 2568/91 [32].

#### 2.3.7. HPLC Analysis of Organic Acids

Twenty grams of sample were melted in 200 mL of hot distilled water, and then decolorized by means of a suitable quantity of carbon powder. The mix was centrifuged at 5000 rpm for 15 min at 5 °C (Centrifuge SL 16 R Thermo Scientific), with subsequent filtration by Whatman *n*° 589/3 filters with a porosity of 150 µm. A collected volume of 30–35 mL was acidified with sulfuric acid (25% w/v) until reaching a pH 2.5. The acidified solution was centrifuged at 8000 rpm for 15 min at 15 °C (Varifuge 20 RS Hereaus Sepatech), in order to further purify the extract from any sediments, pigments, or turbidity. The supernatant obtained was filtered by means of 45 µm syringe filters. HPLC (Spectra system P4000) conditions were as follows: stationary phase Phenomenex Rezex column ROA-ORGANIC ACID H+ at 40°C; mobile phase 0.005 N sulfuric acid solution with a flow of 0.5 mL/min and a 20 μL injection volume; Spectra system UV 1000 detector at 210 nm.

#### 2.3.8. GC Analysis of the Volatile Compounds

Chocolates and related ingredients’ samples were first dispersed in MilliQ water heated at 80 °C, and then centrifuged at 5000 rpm for 15 min at 15 °C (Centrifuge SL 16 R Thermo Scientific) and filtered (Whatman *n*° 595½). Before extraction, magnesium sulfate was added in order to minimize emulsions. Then, 1 mL of sample was substituted with the same volume of internal standard (1-heptanol) at a concentration of 50 ppm. Afterwards, 500 mL of sample was extracted for 6 h with the solvent mixture pentane:dichloromethane (2:1). During extraction, the sample was maintained at 60 °C. At the end it was purified with the addition of anhydrous sodium sulphate and then concentrated by means of Rotavapor (BUCHI Rotavapor R-114) up to a volume of 1 mL. For the determination of volatile compounds, gas chromatography (Autosystem XL Gas Chromatograph Perkin Helmer) with a Flame Ionization Detector (FID) was used, following the chromatographic conditions indicated by Bonvehì [33], by injecting 1 μL of extract. Standard solutions were prepared with pentane:dichloromethane (2:1) as the solvent.

### 2.4. Sensory Analysis

In order to allow a complete sensory description of products and to identify key sensory attributes of chocolates and related ingredients [11] the Quantitative Descriptive Analysis (QDA) [34,35] was applied as a suitable procedure for an assessment of the sensory quality of chocolates and related ingredients during the whole period of storage. No approval from the Human Ethics Committee was required by our institution to perform the sensory analysis in this research.

#### 2.4.1. Determination of the Sensory Profile of Chocolates and Related Ingredients

Sensory properties of the chocolates and related ingredients were monitored at each control time (t0, t1, t2, t3, and t4) under conditions and procedures compliant to ISO standards [36,37,38]. The QDA was carried out using eight assessors with a broad experience in sensory evaluation, as well as interest and availability. The training was performed as reported by Donadini, Fumi, and Lambri [39]. The QDA was composed of the following stages [34,35]: (1) a lexicon generation process and (2) a set of sensory tests designed to quantify the intensity of the sensory descriptors established in the lexicon generation phase on a rating scale. The chocolate and ingredient samples were prepared and individually served to panelists. Three-digit random numbers were assigned to each sample for tracking purposes prior to service. The order of presentation was balanced and randomized across samples, panelists, and replicates, according to a rotated tasting plan [40].

#### 2.4.2. Lexicon Generation Process

In the lexicon generation process, participants were preliminarily asked to name as many sensory characteristics as possible [41,42,43] which they considered important for the descriptive evaluation of chocolate [44,45,46,47,48]. Redundant terms were discussed openly, with the intervention of the panel leader as a moderator. Descriptors cited by at least 30% of the panel were retained and intensively discussed among panelists in an open session until agreement was reached on the final verbal definition. Selected sensory attributes as defined for each sample are reported in Table 1.

#### 2.4.3. Sensory Tests of Chocolates and Ingredients to Rate Sensory Attributes

A set of sensory tests was designed to quantify the intensity of the sensory descriptors that were inserted into the score card on a 9-point scale (anchored at both extremes as “not perceived at all” and “extremely intense”). Panelists were provided with mineral water and unsalted breadsticks to cleanse their palates between samples.

### 2.5. Statistical Analysis

At each storage time starting from production, the sampling proceeded with two independent replications and each analysis was performed in triplicate. Data were subjected to Microsoft Excel 2017 and to Levene’s test to point out the homogeneity of variance among sample subsets. Furthermore, data were analyzed by one-way analysis of variance (ANOVA) with Tukey’s *t*-test at *p* ≤ 0.05 to highlight the significance of the differences among the different storage times (t0, t1, t2, t3, and t4) for equal sample types (CM, C, HP, D, M, and G). The statistics were prepared using IBM SPSS Statistics 20 (IBM Corporation, New York, NY, USA).

## 3. Results and Discussion

### 3.1. Microbiological Analysis

The results of the microbiological analysis were compliant with the limits stated by EC Regulation 2073/2005 [18], as reported in Table 2. Different to other studies focused on microbial evolution in chocolate-based and confectionery products [49], in this study, no meaningful data were obtained. This outlined the microbiological quality of the samples under study, along with the strong hygienic conditions applied in production and storage.

### 3.2. Nutritional Composition and Related Characterization

The sample characterization at t0 along with phenolic compounds, alkaloids, and vitamins is shown in Table 3, while determination of the fatty acid profile, fatty acid composition, and sterol composition is detailed in Table 4. Finally, the organic acids and the volatile compounds are reported in Table 5 and Table 6, respectively.

#### 3.2.1. Chocolates

For D and M chocolates, the content of ash, proteins, and fats (Table 3) confirmed CREA [50] and other authors’ findings [19]. The ash levels were similar between samples and minor at 2%, while proteins were higher in the D sample (at 7.2%) than in the M sample with a content of 6.4% (Table 3). Since D is obtained from the processing of cocoa mass (CM), its results (Table 4) outlined a composition of mainly oleic acid (C18:1), stearic acid (C18:0), palmitic acid (C16:0), and small quantities of linoleic acid (C18:2), confirming what has been reported in the literature [51,52]. As observed by Çakmak et al. [52], oleic acid (C18:1), stearic acid (C18:0), palmitic acid (C16:0), and linoleic acid (C18:2) prevailed in the cocoa mass (CM).

The theobromine (Table 3) at a concentration of about 7.3 mg/g for D and almost 4 mg/g for M agreed with what has been described by Belščak-Cvitanović et al. [53]. Besides, the concentration in D was compliant with Meng et al. [54], who indicated a range of 237–519 mg of theobromine per 50 g portion of dark chocolate. The total soluble polyphenols, with a value of about 2.1 mg GAE/g, and caffeine, with more than 21 mg/100 g in D (Table 3), were similarly remarked upon in a previous study [53].

Citric, oxalic, acetic, malic, lactic, and formic acids were present in all the samples (Table 5), with formic acid in the D sample having a value of 843 ppm, which is greater than in other research [10,55]. The formic acid did not seem to come from the raw materials, but rather from the transformations that occurred during the process: despite its volatility, it was maintained at medium-high levels in the finished chocolates (Table 5). Tartaric acid was completely absent in the chocolates (Table 5), while succinic acid was only found in the M sample with almost a 55-ppm concentration, probably deriving from the added milk.

Finally, considering the volatile compounds detected in the D, M, and G samples, Table 6 shows that D chocolate was characterized by a higher level of pyrazines and benzaldehyde, reaching a value of about 31.5 and 7.5 ppm, respectively, in line with the results of other studies [48,56]. As a matter of fact, pyrazines contribute to 40% of the volatile compounds of roasted cocoa aroma with multiple descriptors. In the study of Liu et al. [48], dark chocolate was characterized by a cocoa flavor with malty, nutty, and toasted notes. Furthermore, Aprotosoaie et al. [57] stated that different cocoa types may exhibit various and specific flavors since the concentration and sensory characters of these compounds vary significantly, depending on the nature and origin of the identified molecules. The literature has stressed a possible microbial fermentation derivation of *Bacillus subtilis* and *Bacillus megatrium*; however, most cocoa and chocolate pyrazines originate in Strecker degradation and Maillard reactions, and need heat and precursors such as aminocinetones, acetoin, and diacetyl [56]. This allows us to conclude that the roasting of both cocoa and hazelnuts has a considerable impact on the aroma profile of the chocolates and related ingredients.

#### 3.2.2. Ingredients

Polyphenols exceeded 0.30 mg GAE/g (Table 3) in HP, as also reported in other research [58,59]. The highest concentrations were found in the C sample, with more than 7 mg GAE/g of polyphenols, as in Belšcak et al. [27], although the literature mentions very variable polyphenol contents, depending on the geographical origin of the beans, degree of maturation, processing, and packaging [7,9,11,29,46]. The content in theobromine and caffeine of the C sample under study (Table 2) was coherent with what was reported by Jalil and Ismail [60], whilst the pH value (Table 3) matched Miller et al.’s results [61], since this sample may be considered as quite alkalized cocoa, showing a pH value greater than 8.00. The alkalization of cocoa powder causes a decrease in the total polyphenols and an increase in pH up to 8.0. Natural cocoa powder has a pH of about 5.3–5.8, while alkalized cocoa powder may be classified according to treatment: light treatment (pH 6.5–7.2), medium treatment (pH 7.2–7.6), and heavy treatment (pH 7.6 and above) [61].

The overall fat content (Table 3) was lower than what was reported by CREA [50], with the most in HP and the least in the C sample. The lipid content is influenced by the geographical origin of cocoa, ranging from 16% to 22% for commercial typologies [6]. The fatty acid composition of C (Table 4) matched what was stated by Elkhori et al. [51]. As for the triglycerides of cocoa butter, our results (Table 4) outlined mainly oleic acid (C18:1), stearic acid (C18:0), palmitic acid (C16:0), and small amounts of linoleic acid (C18:2), as presented in the literature [51], while the Vitamin E content found in C (Table 3), i.e., more than 9%, complied with Lipp et al.’s findings [62]. The saturated, monounsaturated, and polyunsaturated fatty acid content (Table 4) of CM agreed with the values presented by Çakmak et al. [52]. Oleic acid exceeded 30% in all of the samples, reaching almost 83% in the HP sample (Table 3). Moreover, there were other fatty acids (lauric, myristic, myristoleic, pentadecanoic, pentadecenoic, palmitoleic, heptadecanoic, heptadecenoic, linolenic, arachic, eicosadienoic and eicosatrienoic, behenic, and lignoceric) ranging between 0.01% and 1% (Table 5), as found by Bignami et al. [63]. Finally, vitamin E (Table 2) ranged around 300 ppm, confirming what was previously reported [63].

Despite various works being carried out on the lipid fraction of hazelnuts, few data are currently available on the organic acids of HP [63,64,65], which, in our study (Table 5), showed malic as the most abundant acid. Citric, oxalic, and acetic were the only acids detected in the C sample (Table 5). Tartaric and succinic acid were observed in the CM (Table 5), but they were completely absent in the other samples, while formic acid was only present in the HP (Table 4). Succinic acid contained in CM at a concentration of 48 ppm was probably a derived fermentation product. D-L lactic and L-malic acid were present in both CM and HP (Table 5).

The analysis of the aroma compounds led to the identification of the only octanoic acid as off-flavor, which showed the highest amounts in C (Table 6). Among the main contributors to the overall profile of cocoa producing, the greatest impacts to chocolate aroma [48,56], tetramethylpyrazine and trimethylpyrazine, responsible for milk coffee-mocha roasted, nutty, and earthy aromas, respectively, were present, especially in CM (Table 5), whilst phenylacetaldehyde (rosy-like aroma) showed the highest value in HP (Table 5).

### 3.3. Eighteen-Month Evolution of Chocolates and Ingredients: Nutritional and Sensory Changes

**Figure 1:**
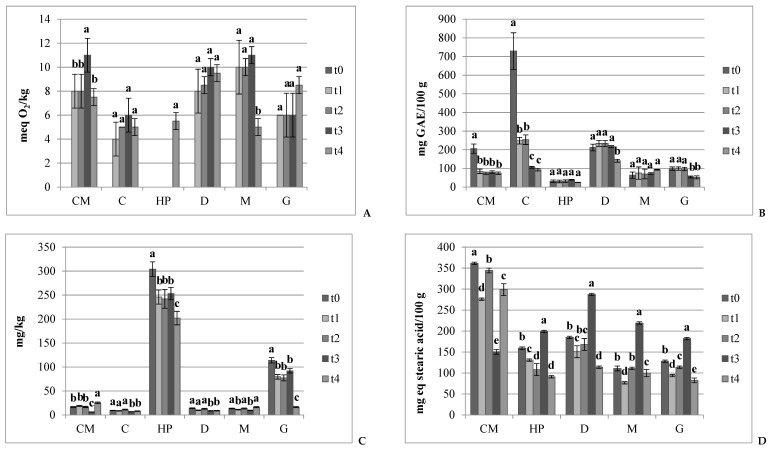
Evolution of the lipid and phenolic matrix at t0, t1, t2, t3, and t4 of (**A**) Peroxide values (expressed as meq of O_2_/kg); (**B**) Unoxidized polyphenol concentration (expressed as mg of GAE per 100 g); (**C**) Vitamin E concentration (mg/kg); (**D**) Acidity values (expressed as mg of stearic acid equivalents per 100 g). Data represent the mean ± SD (*n* = 3). Within each sample, different letters indicate statistically different values among times according to a post-hoc comparison (Tukey’s test) at *p* ≤ 0.05.

As chocolate is a continuous lipid phase, the structural changes in its fat matter may alter volatile release, thus changing the flavor profile of the chocolate [11,14,17,47,48,56,57,66]. As a consequence, chocolates and the related ingredients under study were chemically checked during the 18 months of storage for peroxide and acidity values, polyphenols, and vitamin E [67]. This chemical-physical monitoring was applied with an eighteen-weekly check frequency and outlined for both chocolates and ingredients, and the results are reported in Figure 1.

**Figure 2:**
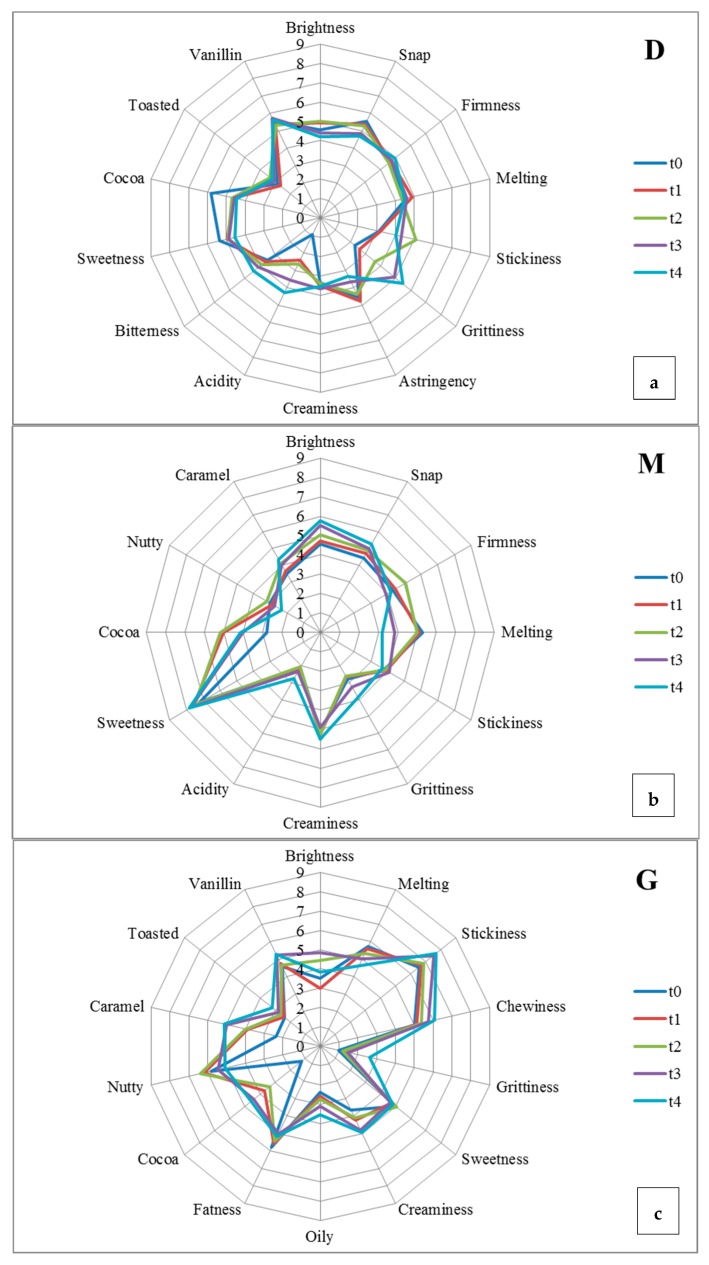
Sensory profile at t0, t1, t2, t3, and t4 of the dark chocolate (**D, subfigure a**), milk chocolate (**M, subfigure b**), and gianduja chocolate (**G, subfigure c**).

**Figure 3:**
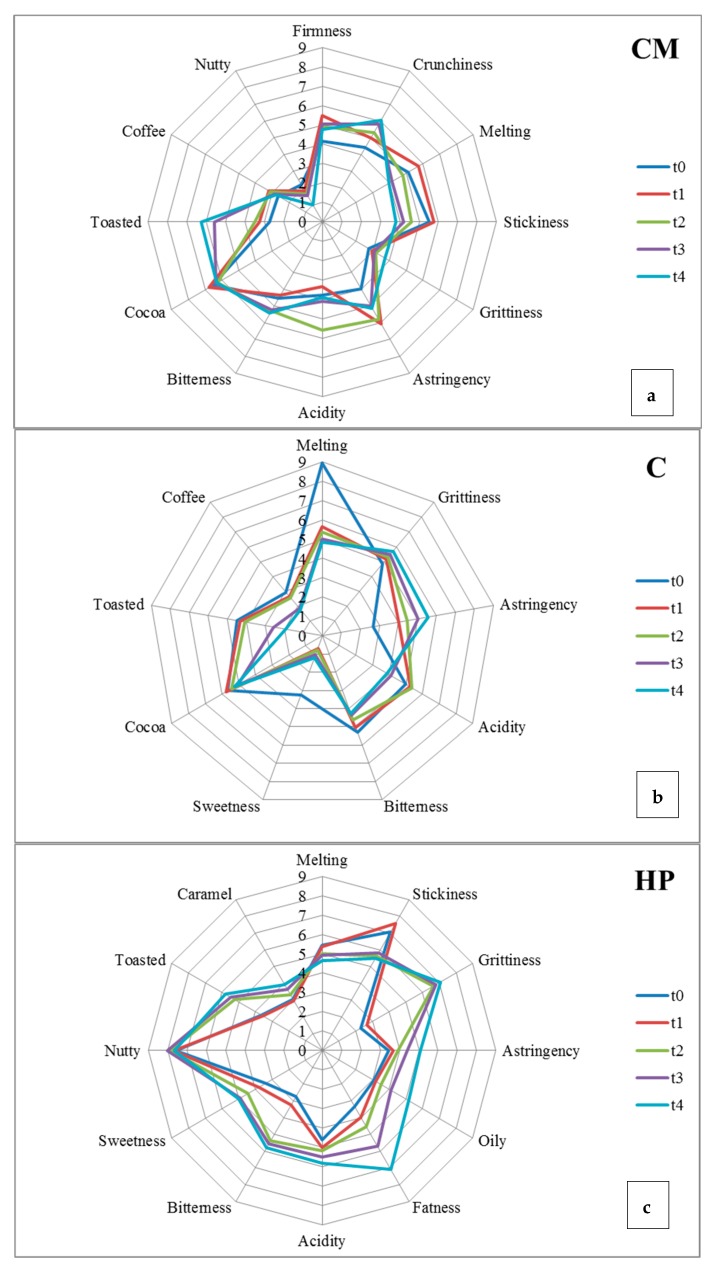
Sensory profile at t0, t1, t2, t3, and t4 of the cocoa mass (**CM**, **subfigure a**), cocoa 22–24 (**C, subfigure b**), and hazelnut paste (**HP, subfigure c**).

On the other hand, the sensory analysis was performed at t0, t1, t2, t3, and t4 by means of QDA profiling that allowed identifying significant trends of increase or decrease in the visual, auditory, mechanical, and flavor perceptions of the samples [11,68]. In the QDA sensory sheet, panelists could indicate descriptors related to the sensory flaws: this was useful for displaying any “anomalies” that arose during storage. The evolution of the sensory attributes of each sample during the eighteen months of conservation is represented in Figure 2 and Figure 3 for ingredients and chocolates, respectively.

#### 3.3.1. Chemo-Sensory Evolution of Chocolates

In the milk chocolate (M sample), brightness and snap increased over time (Figure 2), whereas the intensity of brightness remained constant in D, where the snap became less intense from t0 to t4 (Figure 2a). This was in accordance with Machálková et al. [66], who found a slight deterioration of some mechanical descriptors in the chocolate samples stored at 20 °C. The firmness did not change significantly in D and M samples (Figure 2a,b), while the melting dropped in G and M chocolate (Figure 2c,b). In this regard, Thamke et al. [68] concluded that chocolate with a lower cocoa content was characterized by the greatest melting and creaminess, while the product with the highest cocoa content was characterized as dry dough. This was confirmed by the results from Figure 2c, which show a lower value of creaminess in D chocolate than in M and G samples.

It is interesting to compare the behavior of fat matter with the modification in structural properties observed in chocolate samples and previously described, since the physical state of triglycerides is known to affect the firmness, creaminess, and melting of a chocolate-based product [67]. The alteration of the physical state of fat matter, which is widely observed over time in the storage of fatty food products, is mainly due to rancidity phenomena starting with the hydrolysis of triglycerides giving rise to free fatty acids, which in turn undergo oxidation [69]. This emphasized the role acquired by the monitoring of both acidity and peroxide values that, together with sensory outcomes, in terms of perceived acidity, may help to get quick and consistent information about the fat matter evolution of chocolate-based products during their storage.

Our results evidenced a reduction in the acidity in the first eighteen weeks (t1) (Figure 1D), followed by a fast and similar increase in all the samples after t2 with values of almost 290, 220, and 185 mg eq. stearic acid/100 g in D, M, and G samples, respectively. Afterwards, it fell again until the eighteenth month of storage (t4). Considering the M chocolate (Figure 2b), acidity increased, this was only slightly with respect to the D sample (Figure 2a). Regarding peroxides, the three chocolates maintained a value ranging between 5 and 11 meq of O_2_/kg (Figure 1A). In particular, the results showed no detectable peroxides at the production time (t0), while at the t1, there was a significant increase, especially in M and D chocolate. The fact that in G, the peroxide value remained lower than the other two chocolates, confirms the observations of other authors [70,71], who have attributed this behavior to the phytochemical compounds present in dried fruit, in this case, hazelnuts. As observed for M samples after 18 months (Figure 1A), the peroxide value decreases due to them changing into short-chain aldehydes, or in secondary products deriving from their decomposition [69,72]. Therefore, despite chocolates having a high fat content (Table 3), the lipid oxidation at the end of storage (Figure 1A) was very slow. Both the lyophobic and the lyophilic antioxidants were supposed to perform a continuously protective activity towards fats [14,69] due to their well-known biological effects [3,16,17,27,53,60,61,73,74].

At the end of the eighteen months, the residual content in polyphenols of the chocolates ranged from 50 to 217 mg of GAE per 100 g, highlighting a significant difference between the different types of chocolate. Phenolic substances are involved in the chocolate’s flavor and in the primary sensory characteristics as bitterness and astringency. The analysis (Figure 1B) showed a slight increase in D and M from time t0 to time t1, while in G, the concentration remained constant until t2, and then decreased significantly up to the end of storage (t4), reaching the value of 54 mg GAE/100 g (Figure 1B). Although D chocolate showed an important loss of polyphenols (Figure 1B), it maintained the highest content at the end of storage (t4) when the astringency was perceived to be at a medium level, contrary to M and G chocolate (Figure 2b,c). Studies [11,12,13,66,67] have already observed the depletion of polyphenols in cocoa-based products during storage, correlating this loss with their oxidation in the corresponding quinones, which might lead to increases in bitterness, as outlined in D chocolate (Figure 2a). In the same sample, starting from t2, some panelists marked descriptors related to oxidation as “pungent”, “closed”, “cork”, and “dried fig”. This was in line with Subramaniam [72], who reported that dark chocolate loses some of its chocolate flavor and develops a “stale note” over time. Additionally, she reported that the chocolate develops a stale, “cardboardy” flavor due to oxidative rancidity prior to the onset of fat bloom.

In this regard, results from vitamin E showing a protective antioxidant effect with a reduction potential of 500 mV, comparable to that of epigallocatechin gallate (430 mV) [75], were particularly interesting in the sample derived from hazelnut, i.e., in G chocolate, where a moderate decrease was recorded at t1 and t4 control times (Figure 1C). The concentration in vitamin E detected in G supported its non-significant lipid oxidation (Figure 1A), even if the G polyphenol content was lower than in D and M chocolates (Figure 1B). The G sample (Figure 2c) showed a growth in stickiness, chewiness, creaminess, and grittiness. A drop in the intensity of positive perceptions was also reported by Bomba [76], who noted that the addition of nuts to chocolate shortens its shelf-life, even though the antioxidants in chocolate may be of some benefit to the oil in the nuts. In this regard, the oily attribute continuously rises, even if the fatness perception slightly decreases (Figure 2c). In the M chocolate (Figure 2b), the attributes of “liquorice” and “aged” have also been reported, although at medium levels. Additionally, milk chocolate flavors tend to blend with aging, but extended aging may result in undesirable fruity notes [77]. Liu et al. [48] reported that the typical flavors of milk chocolate are milky, nutty, and caramel with coconut notes, as observed in the present study (Table 5).

#### 3.3.2. Chemo-Sensory Evolution of Ingredients

As regards the evolution of ingredients, the three semi-finished products maintained a peroxide value (Figure 1A) corresponding to a good state of conservation [46], ranging between 4 and 11 meq of O_2_/kg, and even if from t2, the parameter was significantly elevated in the CM. On the contrary, in the HP, the data is not detectable in the first three control times and only at t4 was a value of almost 6 meq of O_2_/kg registered (Figure 1A). This result confirmed the observations of other authors [70], who attributed this behavior to the phytochemical compounds present in the hazelnuts. Indeed, in vitro studies have shown that incubating cells with nut extracts very rich in polyphenols can inhibit oxidative susceptibility [64]. It should be noted that, as observed for C and CM, after 18 months (Figure 1A), the peroxide value may decrease when the hydro-peroxides formed evolve into short-chain aldehydes, i.e., in secondary products deriving from their decomposition. Conversely, the acidity was risen in a linear way only in the HP sample (Figure 3c), while in CM and C, the trend of this attribute was quite fluctuating (Figure 3a and 3b). In detail, the acidity of CM and HP decreased at t1, whereas C had undetectable values at each sampling point (Figure 1D). After the t2 control, the acidity of CM increased, while in HP, it continued to decrease, as also reported by Fardelli [70], who analyzed roasted and natural nuts for nine months of storage.

As well as in chocolates and other studies [12,13,14,15,76], a decrease in the total polyphenol content due to their oxidation was especially observed in CM and C from time t0 to time t1 (Figure 1B) and then from the t2 to t3 for the C sample till the lowest value of 93 mg GAE/100 g. On the other hand, the unoxidized polyphenols remained almost constant for HP throughout the full storage period, ranging between 25 and 39 mg GAE/100 g (Figure 1B). This was quite different from what was observed by Fardelli [70], who reported decreases in polyphenols during nine-month storage of natural and roasted nuts.

The results of vitamin E indicated no significant change during the whole storage time in the HP under examination (Figure 1C), with the exception of a decrease at times t1 and t4. The retained level of vitamin E explains why the HP did not demonstrate detectable lipid oxidation (Figure 1A), even though it had polyphenol content levels that were lower than other samples (Figure 1B). The antioxidant properties of tocopherols come from their ability to donate their phenolic hydrogen to lipid free radicals and to retard the autocatalytic lipid peroxidation processes [78]. Moreover, they show a good stability: after six months of maintenance at room temperature, a 13% decrease in antioxidant activity was already observed [79].

Finally, regarding flavors, an emphasis should be placed on the toasted aroma, whose intensity increased during storage for CM and HP, while it decreased drastically for C. During the eighteen months of storage, in the CM (Figure 3a), the nutty aroma decreased, while cocoa and coffee remained stable. In C, the coffee aroma dropped significantly in the last months of storage, while the cocoa intensity remained constant (Figure 3b). On the contrary, in HP, the caramel aroma increased, in addition to oily and fattiness perception (Figure 3c).

## 4. Conclusions

In the present work, two main groups of cocoa-based foods (chocolates and related ingredients) were investigated by monitoring the evolution of some nutritional components during eighteen months of storage under conditions simulating a point of sale or factory warehouse.

Although the matrices under study contained variable amounts of cocoa butter and hazelnut oil, the evolution of their fats revealed only slow oxidation phenomena. Due to the known effects of catechins, flavonols, and proanthocyanins against lipid peroxidation, the polyphenols measured in chocolates and related ingredients continued to exhibit their protective activity towards fats, even though a general loss in unoxidized polyphenols was observed over time. Finally, despite some alteration of mechanical and structural properties, as well as some losses in aroma, the samples exhibited good sensory scores for the descriptive analysis till the eighteenth month of storage.

Since chocolate, as part of a balanced diet, is becoming a commodity with health and nutritional benefits, these outcomes are really useful for confectionery companies in order to gain detailed information about the state of chocolates and related ingredients which is perceptible by humans and associated with their nutritional composition. Enhancing this knowledge represents a stimulus to people involved in this kind of production, processing, and consumption.

## Figures and Tables

**Table 1:** Descriptors used for sensory profiling.

Attribute	Description	Range and References	Sample(*)
Brightness	Ability to reflect light; luminescence of color, with descriptions ranging from dull to shiny [42]	Low: Dull; dark chocolate 90% cocoa with fat blooming High: Shiny; dark chocolate 90% cocoa	D – M – G
Snap	The noise and force with which the sample breaks or fractures [39]	Low: Gianduja chocolate High: Dark chocolate 90% cocoa	D – M
Firmness	Force required for compressing the sample between molar teeth [45]	Low: Milk chocolate High: Dark chocolate 90% cocoa	MC – D – M
Crunchiness	Easily broken or ruptured Degree to which the sample fractures into pieces on the first bite with the molars [42]	Low: Dark chocolate 90% cocoa High: Milk chocolate	MC
Melting	Chocolate property of melting in mouth while chewing [45] till liquefaction [46]	Low: Dark chocolate 70% cocoa warmed in a microwave oven during 20 s. High: Dark chocolate 70% cocoa warmed in a microwave oven during 40 s.	MC – C – HP – D – M – G
Stickiness	The degree a sample sticks to the palate [42,47]		MC – HP – D – M – G
Chewiness	Length of time required to masticate the sample, at a constant rate of force application, to reduce it to a consistency suitable for swallowing [42]		G
Grittiness	Presence of perceptible particles in the oral cavity. The number of solid particles during mastication [42]		MC – C – HP – D – M – G
Astringency	Mouth drying and/or puckering effect which boosts the production of saliva; perceived between tongue and palate or at the back of the front teeth [42,47]	None: Milk chocolate High: Dark chocolate 90% cocoa	MC – C – HP – D
Oily	The amount of oil left on mouth surfaces [43]		HP – G
Fatness	Surface textural attributes relating to the perception of the quantity or quality of fat in a product [43]		HP – G
Creaminess	The mouth-feel related to the smoothness of the chocolate as related to fat [44]		D – M – G
Acidity	Citric acid (fruit), acetic acid (vinegar), lactic acid (sour milk), and mineral acid (metallic tasting) [42,46]		MC – C – HP – D – M
Bitterness	The taste on the tongue associated with substances such as caffeine and quinine [42]	None: Distilled water High: Caffeine solution at 0.5%	MC – C – HP – D
Sweetness	The taste on the tongue associated with sucrose and other sugars or sweeteners [42]	Low: Sugar solution at 1% High: Sugar solution at 10%	C – HP – D – M – G
Cocoa	The flavor associated with cocoa powder or cocoa beans [43]	Low: Powder cocoa solution at 0.5% High: Powder cocoa solution at 5.0%	MC – C – D – M – G
Toasted/Roasted	Flavor related to cocoa that is very toasted [46] The aroma associated with popcorn or roasted peanut [48]	Low: Dry cocoa seed without toasting. High: Cocoa seed toasted for 3 h	MC – C – HP – D – G
Coffee	The aroma associated with medium-high toasted coffee [39]		MC – C
Nutty	Delicate aroma of indistinguishable nuts without roast. Mixed raw nuts powder (hazelnut, walnut, peanut, and sunflower seeds) [48]		MC – HP – M – G
Caramel	The aroma associated with caramelized sugar [48]	Low: Dry sugar High: Sugar warmed at 120 °C until a brown color	HP – M – G
Vanillin	The aroma associated with vanillin [39]		D – M – G

(*) D=dark chocolate; M=milk chocolate; G=gianduja chocolate; CM=cocoa mass; C=cocoa powder 22–24; HP=hazelnut paste.

**Table 2:** Results from microbiological analysis at the end of storage.

Parameter	CM	C	HP	D	M	G
Total microbial count	<5000	<5000	<5000	<5000	<5000	<5000
Enterobacteriaceae	n.d.	n.d.	n.d.	n.d.	n.d.	n.d.
Coliforms	n.d.	n.d.	n.d.	n.d.	n.d.	n.d.

n.d. = not detectable.

**Table 3:** Characterization at t(0) along with phenolic compounds, alkaloids, and vitamins analyzed in cocoa mass (CM), cocoa 22-24 (C), hazelnut paste (HP), dark chocolate (D), milk chocolate (M), and gianduja chocolate (G). Data represent the mean ± SD (*n* = 3). Within each row, different superscript letters indicate statistically different values among samples according to a post-hoc comparison (Tukey’s test) at *p* ≤ 0.05.

Parameter	CM	C	HP	D	M	G
Humidity (%)	1.06 ± 0.03 ^d^	2.11 ± 0.01 ^a^	0.75 ± 0.01 ^e^	0.70 ± 0.03 ^f^	1.17 ± 0.09 ^c^	1.37 ± 0.00 ^b^
pH	5.51 ± 0.02 ^e^	8.31 ± 0.10 ^a^	5.71 ± 0.01 ^d^	5.70 ± 0.05 ^d^	6.29 ± 0.04 ^b^	6.17 ± 0.03 ^c^
Acidity (mg eq stearic acid/100 g)	361.00 ± 0.01 ^a^	n.d.	159.00 ± 0.01 ^c^	184.91 ± 2.84 ^b^	110.95 ± 5.69 ^e^	128.02 ± 2.84 ^d^
Ash (%)	4.37 ± 0.97 ^b^	11.70 ± 0.04 ^a^	2.05 ± 0.01 ^c^	1.67 ± 0.03 ^e^	1.71 ± 0.01 ^d^	1.65 ± 0.01 ^f^
Protein (%)	14.11 ± 0.63 ^b^	23.33 ± 0.08 ^a^	3.90 ± 0.18 ^f^	7.20 ± 0.01 ^d^	6.40 ± 0.08 ^e^	10.10 ± 0.41 ^c^
Fat matter (%)	35.70 ± 0.04 ^c^	17.90 ± 0.12 ^f^	66.80 ± 1.75 ^a^	23.20 ± 0.30 ^e^	34.20 ± 1.61 ^d^	37.50 ± 2.30 ^b^
Total sugar (%)	0.80 ± 0.00 ^e^	1.20 ± 0.03 ^d^	1.60 ± 0.57 ^d^	45.60 ± 0.31 ^b^	54.70 ± 0.25 ^a^	40.80 ± 1.39 ^c^
Fiber (%) *	12.50 ± 0.06 ^b^	28.03 ± 0.14 ^a^	8.83 ± 0.04 ^c^	7.56 ± 0.04 ^d^	1.60 ± 0.01 ^f^	5.45 ± 0.03 ^e^
* raw fiber (%)	11.10	13.63	n.d.	n.d.	n.d.	n.d.
Phenols (mg Gallic Acid Equivalents /g)	2.06 ± 0.25 ^b^	7.29 ± 0.98 ^a^	0.31 ± 0.07 ^e^	2.12 ± 0.18 ^b^	0.64 ± 0.17 ^d^	0.99 ± 0.09 ^c^
Caffeine (mg/100 g)	65.67 ± 4.97 ^b^	97.69 ± 5.46 ^a^	3.40 ± 0.16 ^e^	21.51 ± 1.04 ^c^	2.06 ± 0.93 ^f^	7.84 ± 2.27 ^d^
Theobromine (mg/g)	6.77 ± 0.68 ^b^	10.07 ± 0.10 ^a^	2.04 ± 0.37 ^d^	7.28 ± 0.59 ^b^	3.97 ± 0.12 ^c^	3.63 ± 0.25 ^c,d^
13-Cis-β-Carotene (ppm)	<0.10 ± 15%	<0.10 ± 15%	<0.10 ± 15%	<0.10 ± 15%	<0.10 ± 15%	<0.10 ± 15%
9-Cis-β-Carotene (ppm)	<0.10 ± 15%	<0.10 ± 15%	<0.10 ± 15%	<0.10 ± 15%	<0.10 ± 15%	<0.10 ± 15%
All-Trans-α-Carotene (ppm)	<0.10 ± 15%	<0.10 ± 15%	<0.10 ± 15%	<0.10 ± 15%	<0.10 ± 15%	<0.10 ± 15%
All-Trans-β-Carotene (ppm)	0.34 ± 15%	<0.30 ± 15%	<0.10 ± 15%	<0.30 ± 15%	0.47 ± 15%	0.30 ± 15%
β-Cryptoxanthin (ppm)	<0.10 ± 15%	<0.10 ± 15%	<0.10 ± 15%	<0.10 ± 15%	<0.10 ± 15%	<0.10 ± 15%
Retinol (ppm)	<0.10 ± 15%	<0.10 ± 15%	<0.10 ± 15%	<5.00 ± 15%	82.00 ± 15%	5.00 ± 15%
Vitamin E (ppm)	6.77 ± 10% ^d^	9.40 ± 10% ^c^	304.00 ± 10% ^a^	8.20 ± 10% ^c^	6.63 ± 10% ^d^	114.00 ± 10% ^b^

**Table 4:** Fatty acid profile, fatty acid composition, and sterol composition expressed in % (as 100 of the total) in cocoa mass (CM), cocoa 22-24 (C), hazelnut paste (HP), dark chocolate (D), milk chocolate (M), and gianduja chocolate (G) at production time (t0).

Fatty acid / Sterol	CM	C	HP	D	M	G
Saturated fatty acids	63.90	61.29	9.49	63.87	65.23	37.67
Monounsaturated fatty acids	32.95	35.34	83.17	32.69	31.60	57.19
Polyunsaturated fatty acids	3.05	3.30	7.24	3.34	3.12	5.10
Trans-oleic fatty acids	<0.01	<0.01	<0.01	<0.01	0.64	0.21
Trans-linoleic fatty acids	<0.01	<0.01	<0.01	<0.01	<0.01	<0.01
Trans-linolenic fatty acids	<0.01	<0.01	<0.01	<0.01	<0.01	<0.01
Trans-palmitoleic fatty acids	<0.01	<0.01	<0.01	<0.01	<0.01	<0.01
C4:0 Butyric	<0.01	<0.01	<0.01	<0.01	0.69	0.23
C6:0 Capronic	<0.01	<0.01	<0.01	<0.01	0.43	0.13
C7:0 Enantiic	<0.01	<0.01	<0.01	<0.01	<0.01	<0.01
C8:0 Caprylic	<0.01	<0.01	<0.01	<0.01	0.28	0.10
C10:0 Capric	<0.01	<0.01	<0.01	<0.01	0.58	0.22
C10:1 Caproleic	<0.01	<0.01	<0.01	<0.01	0.07	<0.05
C12:0 Lauric	<0.01	<0.01	<0.01	<0.01	0.71	0.32
C12:1 Lauroleic	<0.01	<0.01	<0.01	<0.01	<0.05	<0.05
C13:0 Tridecanoic	<0.01	<0.01	<0.01	<0.01	<0.01	<0.01
C13:1 Tridecenoic	<0.01	<0.01	<0.01	<0.01	<0.01	<0.01
C14:0 Myristic	0.10	0.11	<0.05	0.21	2.34	0.98
C14:1 Miristoleic	0.01	<0.01	<0.01	<0.01	0.20	0.08
C15:0 Pentadecanoic	<0.05	<0.01	<0.01	0.05	0.26	0.12
C15:1 Pentadecenoic	<0.01	<0.01	<0.01	<0.01	<0.01	<0.01
C16:0 Palmitic	26.66	27.83	6.61	25.99	26.96	16.57
C16:1 Palmitoleic	0.27	0.28	0.27	0.24	0.51	0.35
C17.0 Eptadecanoic	0.22	0.23	<0.05	0.23	0.38	0.16
C17:1 Eptadecenoic	<0.05	<0.05	0.08	<0.05	0.09	0.08
C18:0 Stearic	35.67	32.08	2.68	36.15	31.53	18.10
C18:1 Oleic	32.68	35.06	82.68	32.40	30.73	56.60
C18:2 Linoleic	2.87	3.10	7.16	3.11	2.83	4.94
C18:3 Linolenic	0.18	0.20	0.08	0.23	0.29	0,16
C20:0 Arachic	1.01	0.90	0.15	1.03	0.90	0,57
C:20:1 Eicosenoic	<0.05	<0.05	0.14	0.05	<0.05	0.08
C22:0 Behenic	0.17	0.14	0.05	0.17	0.16	0.11
C22:1 Erucic	<0.01	<0.01	<0.01	<0.01	<0.01	<0.01
C22:0 Lignoceric	0.07	<0.01	<0.05	0.09	0.08	0.06
Cholesterol	1.00	1.00	0.30	1.30	26.80	14.70
Brassicasterol	<0.10	<0.10	<0.10	<0.10	<0.10	<0.10
2,4-methylene cholesterol	0.30	0.50	0.10	0.20	0.20	0.20
Campesterol	9.00	9.40	4.10	9.60	7.20	6.60
Campestanol	0.20	0.30	0.40	0.20	0.10	0.20
Stigmasterol	25.80	26.10	1.20	24.70	17.80	13.90
Delta-7-campesterol	<0.10	<0.10	<0.10	<0.10	<0.10	<0.10
Delta-5,23-stigmastadienol	<0.10	<0.10	<0.10	<0.10	<0.10	<0.10
Clerosterol	0.80	0.70	0.70	0.70	0.50	0.90
Beta-sitosterol	58.70	56.30	83.40	58.40	44.20	56.70
Sitostanol	0.70	0.70	1.40	0.70	0.40	1.00
Delta-5-avenasterol	2.50	2.50	6.20	2.40	2.00	3.00
Delta-7,9(11)-stigmastadienol	0.20	<0.10	<0.10	<0.10	<0.10	<0.10
Delta-5,24-stigmastanediol	0.40	0.20	0.80	0.80	0.20	0.80
Delta-7-stigmastenol	0.40	0.90	0.70	0.60	0.20	1.00
Delta-7-avenasterol	0.20	0.70	0.80	0.30	0.10	0.50

**Table 5:** Organic acids (mg/kg) in cocoa mass (CM), cocoa 22–24 (C), hazelnut paste (HP), dark chocolate (D), milk chocolate (M), and gianduja chocolate (G) analysed at production time (t0). Data represent the mean ± SD (*n* = 3). Within each row, different superscript letters indicate statistically different values among samples according to a post-hoc comparison (Tukey’s test) at *p* ≤ 0.05.

Organic Acids	CM	C	HP	D	M	G
Ossalic acid	889 ± 27.3 ^b^	1824 ± 33.2 ^a^	195 ± 4.6 ^d^	421 ± 9.7 ^c^	37 ± 0.4 ^f^	79 ± 12.7 ^e^
Citric acid	1765 ± 69.7 ^b^	3640 ± 51.2 ^a^	547 ± 36.1 ^d^	1163 ± 98.5 ^c^	1681 ± 78.5 ^b^	1253 ± 34.7 ^c^
Acetic acid	902 ± 13.4 ^b^	2750 ± 5.8 ^a^	610 ± 7.9 ^c^	109 ± 0.3 ^e^	547 ± 34.6 ^d^	454 ± 78.2 ^d,e^
L-malic acid	444 ± 15.9 ^b^	n.d.	1524 ± 167.4 ^a^	399 ± 2.5 ^c^	194 ± 12.8 ^d^	1388 ± 54.9 ^a^
Lactic acid	570 ± 3.3 ^b^	n.d.	17 ± 0.7 ^e^	194 ± 0.8 ^d^	874 ± 56.8 ^a^	434 ± 23.8 ^c^
Formic acid	n.d.	n.d.	208 ± 16.1 ^b^	843 ± 6.4 ^a^	229 ± 34.1 ^b^	202 ± 5.8 ^b^
Tartaric acid	235 ± 15.7	n.d.	n.d.	n.d.	n.d.	n.d.
Succinic acid	48 ± 0.7 ^b^	n.d.	n.d.	n.d.	54 ± 4.7 ^a^	n.d.

n.d. = not detectable.

**Table 6:** Volatile compounds (mg/kg) in cocoa mass (CM), cocoa 22–24 (C), hazelnut paste (HP), dark chocolate (D), milk chocolate (M), and gianduja chocolate (G) analysed at production time (t0). Data represent the mean ± SD (*n* = 3). Within each row, different superscript letters indicate statistically different values among samples according to a post-hoc comparison (Tukey’s test) at *p* ≤ 0.05.

Volatile Compounds	CM	C	HP	D	M	G	References	Descriptors
2,5-Dimethylpyrazine	n.d.	22.69 ± 2.20 ^a^	5.21 ± 1.54 ^b^	n.d.	0.46 ± 0.07^d^	0.95 ± 0.02 ^c^	0.23–1.69	cocoa, roast nuts
2,6-Dimethylpyrazine	3.32 ± 0.03 ^b^	2.36 ± 0.47 ^c^	12.67 ± 2.58 ^a^	15.94 ± 3.19 ^a^	n.d.	14.10 ± 1.19 ^a^	0.11–0.39	nutty, coffee, green
2,3,5-Trimethylpyrazine	41.75 ± 7.39 ^a^	19.18 ± 3.57 ^b^	3.63 ± 0.45 ^d^	7.38 ± 2.69 ^c^	0.12 ± 0.02 ^f^	0.33 ± 0.17 ^e^	0.21–1.71	cocoa, roast nuts, peanut
2,3,5,6-Tetramethylpyrazine	6.50 ± 1.51 ^a^	4.50 ± 2.11a ^b^	2.03 ± 1.26 ^c^	8.15 ± 1.89 ^a^	1.58 ± 0.11 ^c^	4.53 ± 0.57 ^a,b^	0.52–8.28	chocolate, cocoa, coffee
Benzaldehyde	0.59 ± 0.31 ^d^	3.86 ± 0.12^b^	0.48 ± 0.04 ^e^	7.52 ± 3.50 ^a^	1.80 ± 0.60 ^c^	0.73 ± 0.29 ^d^	0.5–1.89	bitter
2-Acetyl-5-methylfuran	n.d.	5.06 ± 0.92 ^a^	1.43 ± 0.16 ^b^	1.76 ± 0.45 ^b^	0.50 ± 0.14 ^c^	0.43 ± 0.01 ^c^		
2-Phenylacetaldehyde	0.55 ± 0.07 ^c^	2.15 ± 0.75 ^b^	4.02 ± 0.35 ^a^	1.47 ± 1.12b ^c^	2.59 ± 0.54 ^b^	1.55 ± 0.07b ^c^	2–8.90	berry, nutty
α-Terpenilformato	0.16 ± 0.01 ^c^	n.d.	0.72 ± 0.11 ^b^	n.d.	0.71 ± 0.22 ^b^	2.89 ± 0.34 ^a^	0–0.38	herbaceous, citrus
Benzyl acetate	0.37 ± 0.29 ^d^	n.d.	1.92 ± 0.01 ^a^	0.59 ± 0.12 ^c^	0.45 ± 0.04 ^d^	1.32 ± 0.13 ^b^	0–0.033	floral, jasmine
Octanoic acid	0.40 ± 0.29 ^b^	1.93 ± 0.96 ^a^	1.12 ± 0.09 ^a^	1.62 ± 0.02 ^a^	0.61 ± 0.23 ^b^	0.85 ± 0.01 ^a^	0.021–0.37	unpleasant, oily, fatty
2-Acetyl pyrrole	0.18 ± 0.03 ^c^	2.74 ± 1.71 ^a^	1.52 ± 0.31 ^a^	n.d.	0.36 ± 0.01 ^b^	1.58 ± 1.11 ^a^	0.021–0.38	bread, walnut, licorice
3-Hydroxy-2-methylpyridine	n.d.	1.63 ± 0.06 ^b^	1.84 ± 0.32 ^b^	4.90 ± 1.94 ^a^	0.59 ± 0.04 ^d^	0.94 ± 0.06 ^c^	0.14–0.38	wizened
2,3-Dihydro-3,5-dihydro-6-methyl-4-pyrone	n.d.	n.d.	n.d.	n.d.	0.50 ± 0.14	n.d.	0.28–1.87	roasted
3,5-Hydroxy-6-methyl-4-pyrone	n.d.	n.d.	n.d.	n.d.	2.59 ± 0.54	n.d.	0.02–0.37	roasted

n.d. = not detectable.
